# Supplementary material for: The Vitamin D Receptor as a Prognostic Marker in Breast Cancer—A Cohort Study
Source: Nutrients. 2024 Mar 23;16(7):931. doi: 10.3390/nu16070931 (PMC11013402; doi:10.3390/nu16070931)
Supplement: Supplementary file 1 [file nutrients-16-00931-s001.zip › nutrients-2836273-supplementary.pdf]

Supplementary Table S1. Distribution of patient and tumor characteristics, treatment, and events in relation to cytoplasmic VDR intensity.

Cytoplasmic VDR intensity n (%)	All 1018	Negative 62 (7.1)	Weak 208 (23.7)	Moderate 496 (56.5)	Strong 112 (12.8)
**Factor**	**n (%)**	**n (%)**	**n (%)**	**n (%)**	**n (%)**
Age at diagnosis ≥50	816 (80.2)	45 (72.6)	164 (78.8)	404 (81.5)	90 (80.4)
BMI at inclusion ≥25	503 (50.8)	35 (57.4)	109 (52.9)	236 (49.7)	56 (50.1)
Unknown	28	1	2	21	2
Waist circumference ≥80 cm	731 (74.6)	46 (78.0)	163 (79.9)	343 (72.2)	81 (75.0)
Unknown	38	3	4	21	4
Total breast volume ≥850 ml	492 (57.3)	34 (64.1)	111 (61.3)	225 (53.8)	52 (57.1)
Unknown	160	9	27	78	21
Parous	896 (88.0)	55 (88.7)	185 (88.9)	438 (88.3)	94 (83.9)
Ever use of oral contraceptives	722 (71.0)	49 (79.0)	151 (72.6)	340 (68.7)	78 (69.6)
Ever use of menopausal hormone therapy	446 (43.9)	24 (38.7)	82 (39.4)	229 (46.4)	42 (37.8)
Alcohol abstainer	106 (10.4)	9 (14.8)	20 (9.7)	52 (10.5)	14 (12.5)
Smoking current	206 (20.2)	13 (21.0)	39 (18.8)	95 (19.2)	24 (21.4)
Vitamin D supplements	103 (10.2)	8 (12.9)	15 (7.2)	55 (11.1)	9 (8.0)
Unknown	7				
Season of operation					
Winter (Jan-Mar)	251 (24.7)	17 (27.4)	53 (25.5)	125 (25.2)	23 (20.5)
Spring (Apr-Jun)	303 (29.8)	20 (32.3)	53 (25.5)	153 (30.8)	36 (32.1)
Summer (Jul-Sep)	177 (17.4)	11 (17.7)	38 (18.3)	79 (15.9)	21 (18.8)
Fall (Oct-Dec)	287 (28.2)	14 (22.6)	64 (30.8)	139 (28.0)	32 (28.6)
Detection mode (45-74yrs)					
Screening	569 (66.2)	27 (57.4)	103 (59.9)	276 (65.6)	70 (72.9)
Invasive tumor size					
>20 mm*	275 (27.0)	22 (35.5)	60 (28.8)	124 (25.0)	35 (31.3)
Lymph node status					
Positive	389 (38.2)	26 (41.9)	74 (35.6)	208 (41.9)	43 (38.4)
Nottingham grade					
III	257 (25.2)	41 (66.1)	89 (42.8)	95 (19.2)	13 (11.6)
Histological type					
Ductal	824 (80.9)	54 (87.1)	183 (88.0)	410 (82.7)	83 (74.1)
Lobular	116 (11.4)	1 (1.6)	9 (4.3)	51 (10.3)	23 (20.5)
Other/mixed	78 (7.7)	7 (11.3)	16 (7.7)	35 (7.1)	6 (5.4)
ER status pos (>10%)	894 (87.8)	21 (33.9)	159 (76.4)	478 (96.4)	111 (99.1)
PgR status pos (>10%)	723 (71.0)	13 (21.0)	125 (60.1)	392 (79.0)	90 (80.4)
HER2 amplified	110 (11.5)	8 (13.1)	34 (16.8)	56 (11.5)	9 (8.4)
Unknown	63	1	6	8	5
Triple negative	74 (7.3)	34 (56.6)	29 (13.9)	5 (1.0)	0 (0.0)
Unknown	7	2	0	0	0
Final type of operation					
Mastectomy	410 (40.3)	35 (56.5)	127 (61.1)	294 (59.3)	66 (58.9)
Chemotherapy	258 (25.3)	36 (58.1)	75 (36.1)	102 (20.6)	21 (18.8)
Radiotherapy	644 (63.3)	44 (71.0)	129 (62.0)	309 (62.3)	75 (67.0)
Herceptin	73 (7.2)	5 (8.1)	22 (10.6)	31 (6.3)	6 (5.4)
Endocrine Therapy**					
Tamoxifen	572 (64.0)	19 (90.5)	114 (71.7)	294 (61.5)	74 (66.7)
Aromatase Inhibitor	371 (41.5)	11 (52.4)	68 (42.5)	202 (42.3)	48 (43.2)
Event					
Any breast cancer event	195 (19.2)	9 (14.5)	45 (21.6)	108 (21.8)	11 (9.8)
Death	188 (18.5)	13 (21.0)	40 (19.2)	99 (20.0)	15 (13.4)

Unknown presented when exceeding 0.5%. Percentages does not include missing categories.
*Or muscular or skin involvement (pT2/3/4). **Out of ER-positive n=894.

Supplementary Table S2A. Distribution of patient and tumor characteristics, treatment, and events in relation to nuclear membrane VDR in patients with ER positive tumors VDR.

**Nuclear membrane VDR n (%)**	**Negative 555 (7.1)**	**Positive 214 (23.7)**	
**Factor**	**n (%)**	**n (%)**	***P*-value***
Invasive tumor size			
>20 mm*	154 (27.7)	48 (22.4)	0.107
Lymph node status			
Positive	234 (42.3)	79 (36.9)	0.156
Nottingham grade			
III	128 (23.1)	23 (10.7)	<0.001
Histological type			
Ductal	454 (81.8)	175 (81.8)	
Lobular	59 (10.6)	24 (11.2)	0.985
Other/mixed	42 (7.6)	15 (7.0)	
PgR status pos (>10%)	436 (78.6)	179 (83.6)	0.180
HER2 amplified	53 (9.9)	9 (4.2)	0.010
Final type of operation			
Mastectomy	229 (41.3)	84 (39.3)	0.664
Chemotherapy	114 (20.5)	40 (18.7)	0.172
Radiotherapy	340 (61.3)	138 (64.5)	0.593
Herceptin	34 (6.1)	9 (4.2)	0.141
Event			
Any breast cancer event	116 (20.9)	28 (13.1)	0.081
Death	109 (19.6)	23 (10.7)	0.022

* *P*-value adjusted for time between surgery and staining.

Supplementary Table S2B. Breast cancer free interval and overall survival in relation to ER/VDR^{num} status.

Nuclear membrane VDR level	Included analyses	inTotal n	Events n	HR[1] (CI 95%)	HR[2] (CI 95%)	HR[3] (CI 95%)
**Breast cancer free interval**						
ERnegVDR^{num}neg	Complete case*	108	29	ref	ref	ref
ERnegVDR^{num}pos		1	0	not reliable	not reliable	not reliable
ERposVDR^{num}neg		555	116	0.64 (0.43-0.96)	0.75 (0.47-1.18)	1.27 (0.70-2.30)
ERposVDR^{num}pos		214	214	0.42 (0.25-0.70)	0.55 (0.30-0.98)	0.89 (0.45-1.76)
ERnegVDR^{num}neg	All Included**	119	31	ref	ref	ref
ERnegVDR^{num}pos		4	0	not reliable	not reliable	not reliable
ERposVDR^{num}neg		645	131	0.67 (0.46-1.00)	0.81 (0.52-1.26)	1.38 (0.79-2.41)
ERposVDR^{num}pos		250	33	0.47 (0.28-0.77)	0.62 (0.35-1.09)	1.00 (0.98-1.01)
**Overall survival**						
ERnegVDR^{num}neg	Complete case*	108	35	ref	ref	ref
ERnegVDR^{num}pos		1	0	not reliable	not reliable	not reliable
ERposVDR^{num}neg		555	109	0.50 (0.34-0.73)	0.52 (0.34-0.81)	0.77 (0.42-1.39)
ERposVDR^{num}pos		214	23	0.29 (0.17-0.49)	0.36 (0.20-0.65)	0.51 (0.25-1.04)
ERnegVDR^{num}neg	All Included**	116	36	ref	ref	ref
ERnegVDR^{num}pos		7	1	not reliable	not reliable	not reliable
ERposVDR^{num}neg		646	125	0.52 (0.35-0.76)	0.54 (0.35-0.83)	0.81 (0.46-1.43)
ERposVDR^{num}pos		249	26	0.30 (0.18-0.50)	0.36 (0.20-0.64)	0.54 (0.27-1.06)

*Only patients with no missing values included in analyses. **Multiple imputation used for missing values, all patients in the cohort included in analyses. [1] Model 1: Adjusted for time between surgery and staining. [2] Model 2: Adjusted for time between surgery and staining, age, tumor size, node status, grade III. [3] Model 3: Adjusted for time between surgery and staining, age, tumor size, node status, grade III, BMI, smoking, adjuvant treatment.
